# Literary Gossip and Record

**Published:** 1861-07

**Authors:** 


### Art. XIII.—LITERARY GOSSIP AND RECORD.

In the management of the different Medical Libraries of the Metropolis, in no single instance has the importance been recognised of one of them at least containing a complete series of English medical publications—that is to say, complete from and since the institution of the Library, and as perfect as possible anterior to that period. The most perfect collection of English medical literature is that in the Library of the British Museum, and supposing that that collection should be destroyed, the loss would be irreparable. In the prospect, therefore, of such a contingency, infinitesimal though we hope it may be, the subject is one worthy of the consideration of the managing bodies of medical libraries. But on mere bibliographical grounds alone, we think that at least one of our libraries should make it a rule to purchase every English medical work as published, and should strain every nerve to perfect its collection of national medical literature. The bibliographical importance of a work cannot always be determined at the time of publication, and it is a common matter of experience, that works thought of little moment then, become subsequently of value. Thus, for example (and not a solitary one, by the way), a short time ago we had occasion to refer in haste to the curious edition of the works of the celebrated Dr. John Brown, by Dr. Beddoes, and found that there is actually no copy either in the Library of the British Museum, that of the Medico-Chirurgical Society, or that of the College of Surgeons. Our engagements at the time debarred us from extending our search to other libraries, but, strange to say, we ascertained that there was in the British Museum Library the *German Translation of the book we sought*, as well as the edition of Dr. Brown's works, brought out chiefly in consequence of the supposed injustice done to him and his writings by Dr. Beddoes. This edition we also found in the Medico-Chirurgical and College of Surgeons' Libraries. The only mode of escaping such unfortunate shortcomings for the future, is by the purchase by at least one Medical Library of every English medical work as published, irrespective of its estimated literary or scientific value at the time. Such a proposition is by no means so formidable as it would at first appear, for the actual annual pecuniary cost of these works, including pamphlets, is by no means very large, and, we believe, quite within the limits of the resources commanded at least by the Medico-Chirurgical Society, without affecting the purchases of foreign works. To this Society, indeed, we rightly look as the body most fitted to have respect to the bibliographical credit of the profession, and the pride already felt by the Fellows in their noble Library, ought to induce them to perfect its character as much as possible.

But the need for as perfect a Medical Library as is practicable to be obtained, apart from the National Library, is beginning, from other causes, to make itself sensibly, nay painfully felt, among literary medical men, who at the same time (as is the rule) are engaged in the practice of their profession. To them the Library of the British Museum is becoming a huge bugbear, and, at the best, a thing of scanty utility. For, in consequence of the crowds of readers, consequent upon the increased space provided for them, it frequently happens that an hour is spent (omitting altogether the time occupied in hunting for the book in the Catalogues) before a work wanted can be obtained, and rarely is a book procured under a period of twenty minutes. Thus, to the medical man in active practice, extended reading in the National Library is out of the question, and he is to a great extent deprived of its advantages, except in so far as he can avail himself of the Reference Library. But even supposing that the objection of delay be overcome, it not unfrequently happens that in spite of the splendid collection of medical works in the National Library, for sundry reasons not needful to detail here, the medical reader is obliged, in the pursuit of a given course of literary research, to circulate among the different Medical Libraries, hunting for this thing here and that thing there, at an inordinate expenditure of time and temper.

Now we do not for a moment imagine that these evils will, or can ever, be entirely eradicated, but we are certain that they might be very materially modified, if the managers of any one of the great Medical Libraries, would but consider that it was their duty to perfect the Library under their control at any cost. It may be that the readers of the present generation might not feel very sensibly the advantages arising from such a notion of duty being carried into effect, but the next most assuredly would—a sufficiently honourable incentive we opine.

We appeal, therefore, to the Council and Fellows of the Medico-Chirurgical Society; and now that they are possessed of the idea that their Society is not doing quite so much for the scientific credit of medicine as it might do, we would wish them to become also possessed of the idea that they are not doing as much for the literary credit of the healing art in this kingdom as they might do.

